# Single Oral Acute Toxicity of Banhasasim-Tang and Its Antiobesity Effect on Diet-Induced Obese Mice and 3T3-L1 Adipocytes

**DOI:** 10.1155/2018/3865434

**Published:** 2018-08-29

**Authors:** Sae-Rom Yoo, Soo-Jin Jeong, Mee-young Lee, Hyeun-Kyoo Shin, Chang-Seob Seo, Hyekyung Ha

**Affiliations:** ^1^Herbal Medicine Research Division, Korea Institute of Oriental Medicine, Daejeon 34054, Republic of Korea; ^2^Clinical Medicine Division, Korea Institute of Oriental Medicine, Daejeon 34054, Republic of Korea

## Abstract

We had tested antiobesity effect of 52 traditional herbal formulas in 3T3-L1 adipocyte, and Banhasasim-tang (BHSST) was chosen as one of the effective medications to inhibit triglyceride accumulation. We investigated the antiobesity effect of BHSST on 3T3-L1 adipocytes and high-fat diet- (HFD-) induced obese mice. In addition, we evaluated the acute toxicity of BHSST in Sprague Dawley (SD) rats. Differentiated 3T3-L1 cells were treated with various concentrations of BHSST for 8 days. Accumulated triglyceride level and the expressions of adipogenesis-related genes and proteins were subsequently investigated. To evaluate the single oral toxicity of BHSST, the SD rats of each sex were administered a single dose (5000 mg/kg) of BHSST via oral gavage; the control group received vehicle only. After a single administration, the mortality, clinical signs, gross findings, and body weight were monitored for 15 days. Male C57BL/6J mice were fed HFD for 4 weeks to induce obesity and randomly received 50 mg/kg of Orlistat (n=12, OR), 200 mg/kg of BHSST (n=12, B200), and 1000 mg/kg of BHSST (n=12, B1000) for another 8 weeks. BHSST suppressed the triglyceride contents and lipid accumulation in a dose-dependent manner in 3T3-L1 adipocytes. BHSST also downregulated the adipogenesis-related gene levels and protein expression compared with those in undifferentiated adipocytes. In a single oral dose toxicity study, there was no adverse effect on mortality, clinical signs, body weight changes, and gross findings in the treatment group. HFD-fed mice treated with BHSST showed significantly reduced body weight gain, food efficiency ratio, and white adipose tissue weight. The medial lethal dose (LD50) of BHSST was 5000 mg/kg/day body weight for each sex in the rats. BHSST decreased the body weight gain in HFD-fed obese mice and inhibited triglyceride accumulation via a cascade of multiple factors at the mRNA and protein levels in 3T3-L1 adipocytes.

## 1. Introduction

Obesity is a result of chronic energy imbalance, which causes several metabolic disorders such as hyperglycemia, hypertension, and insulin resistance [1]. The worldwide prevalence of obese and overweight individuals has increased more than threefold since 1975. In 2016, 1.9 billion adults over the age of 18 and 41 million children under the age of 5 were overweight or obese [2]. Along with an increasing number of overweight or obese people, the market for weight-loss products has been rapidly growing.

The use of herbal medicine to manage weight control has received much attention in recent decades. Studies have shown that various herbal medicines are effective for the treatment obesity in clinical trials [3, 4] and their underlying mechanisms or components have been elucidated through in vitro and in vivo experiments [5–7]. Although several herbal medicines have shown promising effects, certain medicines are poorly understood in regard to contamination, toxic dosage, and undesirable adverse reactions. Thus, to determine the safety and efficacy of herbal medicine, it is a necessary factor to develop antiobesity drugs.

Banhasasim-tang (BHSST, Banxiaxiexin-Tang in Chinese and Hangeshashin-to in Japanese), a traditional herbal formula, has been used for centuries in Korea to improve gastrointestinal symptoms such as heartburn, gastritis, and gastric ulcer [8]. Recent pharmacological studies have reported that BHSST has protective effects on inflammation [9, 10] and diabetes [11, 12] as well as gastrointestinal symptoms [13–16]. Despite the various effects of BHSST, few studies have explored its antiobesity effect and toxicity. We had tested antiobesity effect of 52 traditional herbal formulas in 3T3-L1 adipocyte, and BHSST was chosen as one of the effective medication to inhibit triglyceride accumulation.

The aim of the present study was threefold: (1) to determine the effect of BHSST on adipogenesis in 3T3-L1 adipocytes, (2) to assess the acute toxicity of BHSST in Sprague Dawley (SD) rats, and (3) to examine whether BHSST exerts an antiobesity effect on HFD-induced obese mice.

## 2. Materials and Methods

### 2.1. Preparation of BHSST

The eight herbal components were purchased from Kwangmyungdag (Ulsan, South Korea) (Table 1). The origin of eight medicinal herbs was confirmed taxonomically by Professor Je-Hyun Lee, Dongguk University, Gyeongju, Republic of Korea. A ground herbal mixture of 5.0 kg was extracted in a 10-fold volume of water at 100°C for 2 h under pressure (1 kgf/cm^2^) using an electric extractor (COSMOS-660; Kyungseo Machine Co., Incheon, Korea). After filtration through a standard sieve (No. 270, 53 *μ*m) the solution was freeze-dried (Innova^®^ U725 Upright Freezer, Eppendorf, Hamburg, Germany). Lyophilized BHSST extract was dissolved in DBPS (Dulbecco's phosphate buffered saline) and subsequently filtered through a 0.2 *μ*m pore size membrane filter. Voucher specimens (2012-KE-38-1~KE38-8) have been deposited at the Herbal Medicine Research Division, Korea Institute of Oriental Medicine. HPLC analysis data for the quality control of BHSST have been provided in a previous report [9]. Briefly, an established HPLC-PDA method was applied for simultaneous analysis of the nine compounds—baicalin, wogonoside, baicalein, wogonin, glycyrrhizin, liquiritin, berberine, coptisine, and palmatine—in the BHSST extract.

### 2.2. Cell Culture and Differentiation

The 3T3-L1 cells were obtained from the American Type Culture Collection (ATCC, CL-173, MD, USA) and maintained in DMEM supplemented with 10% new born calf serum and 1% penicillin–streptomycin (Thermo Fisher Scientific, MA, USA). After reaching a confluent state, the cells were differentiated with DMEM (Thermo Fisher Scientific) containing 10% fetal bovine serum (FBS), isobutylmethylxanthine, dexamethasone, and insulin (MDI) for 48 h. Subsequently, the medium was switched to DMEM containing 10% FBS and 1 *μ*g/mL insulin after 2 days and later changed to DMEM containing 10% FBS for an additional 4 days. The cells were treated with BHSST every 2 days. GW9962 (Sigma Aldrich, MO, USA) is a peroxisome proliferator-activated receptor-gamma (PPAR-*γ*) antagonist, which was used as a positive control in the present study.

### 2.3. Cytotoxicity Assay

The 3T3-L1 preadipocytes were induced with MDI in the presence or absence of BHSST as describe above. The adipocytes were incubated in CCK-8 solution (Dojindo Molecular Technologies, Inc., MD, USA) for 1 h. After incubation, the optical density was measured using the Benchmark plus microplate reader (Bio-Rad Laboratories, Inc., Hercules, USA).

### 2.4. Oil Red O Staining

Oil Red O staining was assayed according to a modified method [17, 18]. The adipocytes were fixed with 10% formalin for 1 h at room temperature and washed with 70% ethanol and PBS. The cells were stained with Oil Red O (Sigma Aldrich) solution for 30 min. After washing, images were captured by using Olympus CKX41 inverted microscopy (Olympus Co., Tokyo, Japan).

### 2.5. Triglyceride Quantification Assay

The accumulated triglyceride (TG) levels were measured using a commercial kit (BioVision Inc., CA, USA). Briefly, the TG levels were converted to free fatty acids and glycerol by lipase, and oxidized glycerol was subsequently measured by a coupled enzymatic reaction using a microplate reader.

### 2.6. Glycerol-3-Phosphate Dehydrogenase (GPDH) Activity Assay

GPDH activity was measured by using a commercial kit (TaKaRa Bio Inc., Shiga, Japan) according to the manufacturer's instructions. Briefly, we lysed differentiated cells and then collected the supernatants. The supernatants were mixed with substrate solution and the decrease in absorbance due to NADH oxidation was monitored.

### 2.7. Leptin Immunoassay

Leptin production was determined using a commercial kit (R&D Systems, Inc., MN, USA), according to the manufacturer's instructions. The culture supernatants were reacted with a polyclonal antileptin antibody and leptin conjugated to horseradish peroxidase (HRP). After reacting with substrate solution, the change in absorbance was assessed using a microplate reader as described above.

### 2.8. Western Blotting

Cells were lysed with lysis buffer containing protease inhibitor (Roche Diagnostics GmbH, Mannheim, Germany). The protein concentrations were determined by using Bradford reagent (Bio-Rad Laboratories Inc.). The proteins were resolved by SDS-PAGE gels and transferred to polyvinylidene difluoride membranes (Millipore, MA, USA). The membranes were blocked with 5% (w/v) nonfat dry milk in Tris-buffered saline containing Tween 20 (TBST) buffer and later incubated with primary antibody for 24 h at 4°C. After 24 h, the membranes were washed with TBST buffer and incubated with HRP-conjugated secondary antibody for 1 h at room temperature. The immunoreactive bands were developed with ECL reagent (Thermo Fisher Scientific).

### 2.9. RNA Isolation and RT-PCR

Total RNA was prepared with a commercial kit (Intron Biotechnology, Sung-nam, South Korea). Isolated RNA was reverse-transcribed to cDNA and subjected to PCR with rTaq DNA polymerase (ELPIS Biotech Inc., Daejeon, South Korea). The PCR conditions were 22–28 cycles at 94°C for 30 sec, 50–60°C for 1 min, and 72°C for 1.5 min. The PCR products were separated by electrophoresis on 1.5% agarose gels and visualized by ChemiDOC™ XRS (Bio-Rad Laboratories Inc.).

### 2.10. Animals and Experimental Design

To establish the safety information of BHSST, five-week-old SD rats of each sex were purchased from Orient Bio Co., Ltd. (Seongnam, South Korea) and housed under controlled temperature (23±3°C) and humidity (50±10%) with a 12 h light/dark cycle, 10–20 air changes per hour, and a light intensity of 150–300 Lux. After one week of adaptation, the rats were randomly divided into the 5000 mg/kg/day of BHSST-treated group (n=5/each of sex) and the vehicle control group (n=10) based on the latest body weight by using the Pristima system (Version 6.4.1, Xybion Medical System Co., NJ, USA). The rats were fed a rodent diet (Catalog No. 5053, PMI Nutrition International LLC., MO, USA) and sterilized tap water* ad libitum*. The rats were fasted for 12 h before oral administration and fed rodent diet after 3-4 h following the administration. Mortality, clinical sign, and body weight were recorded before BHSST treatment (day 1) and on days 2, 4, 8, and 15 after treatment. After 15 days, all rats were euthanized by carbon dioxide and the gross findings of tissues were observed with naked eyes. Acute toxicity protocol was approved by the Institutional Animal Care and Use Committee of Korea institute of Toxicology (approval number: JG14009) according to the “Guidelines for Toxicity Tests for Drugs and Related Materials, Document #2015-82” as prepared by the Korean Ministry of Food and Drug Safety.

To examine whether BHSST exerts antiobesity effect, five-week-old C57BL/6J male mice were purchased from Dae Han Bio Link Co. (Eum-seong, South Korea) and housed at a constant temperature (21±1°C) and humidity (45±5°C) with 12 h light/dark cycle. After one week of adaptation, the mice were randomly divided into 5 groups (n=12/group). The mice were fed a high-fat diet (HFD; n=48, 45% fat, and D12451, Research Diet Inc., NJ, USA) for 4 weeks to induce obesity. High-fat-induced obese mice then received Orlistat (OR; 50 mg/kg) or 200 mg/kg of BHSST (B200) or 100 mg/kg of BHSST (B1000) for another 8 weeks. A control diet group (ND; n=12, D12450B, Research Diet Inc.) was fed a 10% fat diet for 12 weeks. Orlistat and BHSST were suspended in sterilized 0.5% CMC and administered 10 ml/kg of body weight. Body weight was measured twice a week. Orilstat, a lipase inhibitor, is the only approved antiobesity drug for long-term treatment, which was used as a positive control in the present study. After 12 weeks, the mice were fasted for 10 h and later then euthanized by exsanguination from the postcaval vein and aorta. The animals were maintained in accordance with the National Animal Welfare Law of Korea and approved by the Chungbuk National University Institutional Animal Care and Use Committee (CBNUA-875-15-02).

### 2.11. Measurement of Hepatotoxic Biomarkers in Plasma

Plasma levels of aspartate transaminase (AST) and alanine transaminase (ALT) were measured using by commercial enzymatic kits (BioVision Inc.).

### 2.12. Measurement of Plasma Lipids

Plasma TG (ASAN Pharmaceutical, Seoul, South Korea), total cholesterol, and HDL-cholesterol (BioVision Inc.) levels were enzymatically measured using a commercial kit.

### 2.13. Histopathological Analysis

Inguinal white adipose tissue was fixed with 4% paraformaldehyde and embedded in paraffin for staining with hematoxylin and eosin (H&E). The stained tissue was observed using an Olympus BX51 (Olympus Co.) at a magnification of x200 and photographed with MetaMorph Offline version 7.7.0.0 image analysis software (Molecular Devices, LLC., CA, USA).

### 2.14. Statistical Analysis

All data are presented as means ± the standard error of the mean (SEM). For the in vitro data, significant differences were assessed by one-way ANOVA and post hoc Tukey's multiple comparison test. For in vivo data with C57/BL6J mice, group differences were assessed by one-way ANOVA and post hoc Tukey's multiple-range test by using SYSTAT (Systat Software Inc., IL, USA). For in vivo data with SD rats, the t-test was used to assess the homogeneity of variance. When data have equal variance, group differences were assessed by a t-test and Dunnett's post hoc test using the Path/Tox system (Xybion Medical System Co., NJ, USA). When the data variances are not equal, group differences were assessed by the Kruskal-Wallis test and Dunn's post hoc rank sum test.

## 3. Results

### 3.1. BHSST Inhibits Adipogenesis and Modulates Adipogenesis-Related Factors in 3T3-L1 Adipocytes

**Figure 1:**
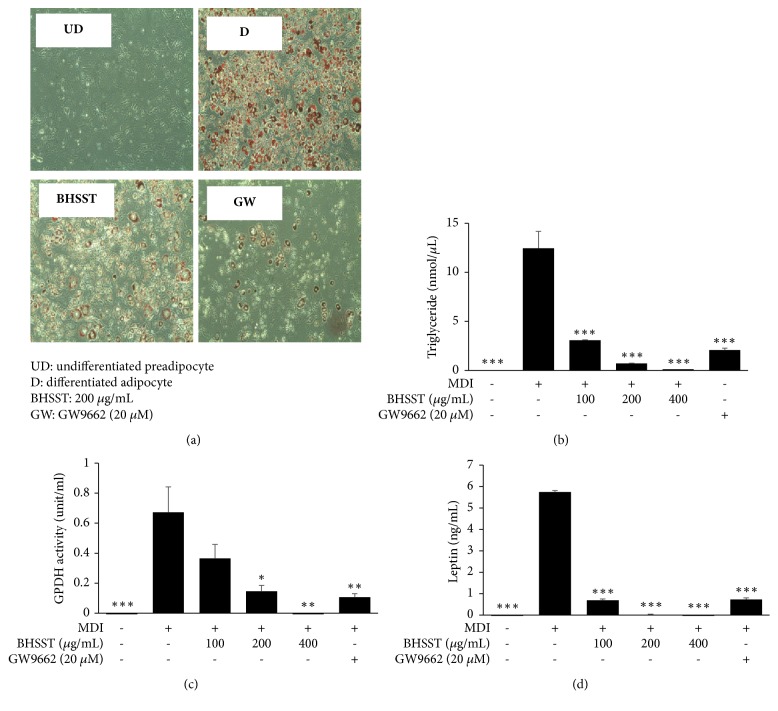
Effect of BHSST on (a) fat accumulation (at 200 x of magnification), (b) GPDH activity, (c) accumulated triglyceride content, and (d) leptin production in mature 3T3-L1 adipocytes. Data are presented as the means ± SEM. ^*∗*^*p* < 0.05 and ^*∗∗∗*^*p* < 0.001 are compared with the differentiated group. GW9662 was used as a positive control. MDI: isobutylmethylxanthine, dexamethasone, and insulin; GPDH: glycerol-3-phosphate dehydrogenase.

No significant toxicity was observed at concentrations up to 500 *μ*g/ml of BHSST in adipocytes (data not shown). Thus, all experiments with 3T3-L1 adipocyte were performed under 500 *μ*g/ml of BHSST. Following the differentiation of preadipocytes to adipocytes, several lipid droplets were markedly observed (Figure 1(a)). However, after BHSST treatment, lipid droplets were less observed in adipocytes compared with untreated adipocytes. Consistent with the morphological observation, GPDH activity and TG content were significantly increased in adipocytes (Figures 1(b) and 1(c)). However, BHSST treatment inhibited the GPDH activity and TG content in a dose-dependent manner compared with untreated adipocytes. In addition, BHSST treatment significantly decreased leptin production (Figure 1(d)).

**Figure 2:**
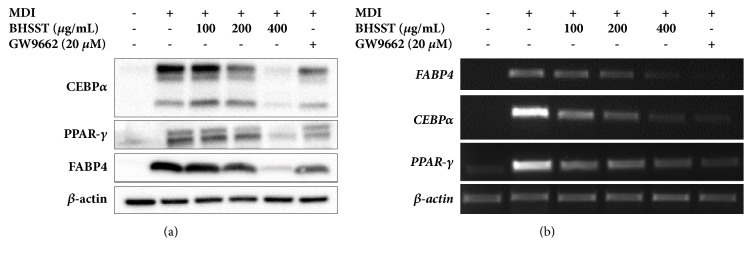
Effect of BHSST on expression of mRNA and protein involved in adipogenesis in 3T3-L1 adipocytes. 3T3-L1 adipocytes were exposed to various concentrations of BHSST during the differentiation period. GW9662 was used as a positive control. MDI: isobutylmethylxanthine, dexamethasone, and insulin.

As shown in Figure 2(a), differentiation increased the protein levels of PPAR-*γ* and CCAAT/enhancer binding protein-alpha (C/EBP-*α*), key adipogenic regulators. Western blotting revealed that BHSST decreased the levels of PPAR-*γ* and C/EBP-*α* proteins compared with those in untreated adipocytes (Figure 2(a)). BHSST also decreased the proteins level of fatty acid binding protein 4 (FABP4) corresponding with PPAR-*γ* expression. Consistent with the immunoblotting results, BHSST downregulated the mRNA expression of PPAR-*γ*, C/EBP-*α*, and FABP4 in adipocytes (Figure 2(b)).

As an antagonist of PPAR-*γ*, GW9662 prevented adipogenesis and decreased adipogenesis-related protein and mRNA expression (Figures 1 and 2).

### 3.2. BHSST Did Not Show Adverse Effects on SD Rats in the Acute Toxicity Test

**Figure 3:**
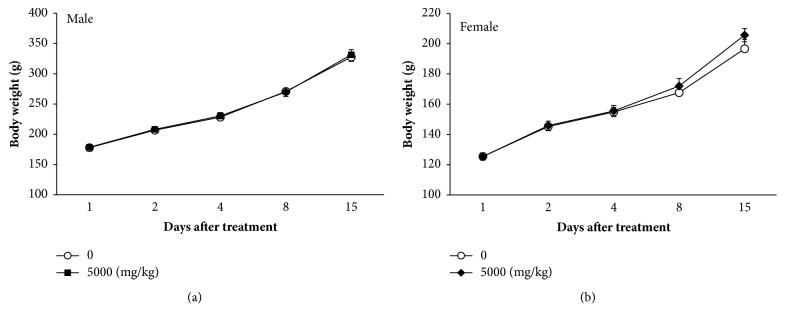
Effect of BHSST on body weight changes in (a) male or (b) female rats. Data are presented as the means ± SEM.

No death occurred in any of the animals over the 15 days of the experimental period (Table 2). A loss of fur was observed in one female rat in the vehicle control group (Table 2). No gross findings in internal organs were observed in any of the animals (Table 2). During the experimental period, the rats gained weight in accordance with their age. None of the rats showed any significant body weight changes after a single oral administration of BHSST (Figure 3).

### 3.3. BHSST Prevents Fat Accumulation in Diet-Induced Obese Mice

**Figure 4:**
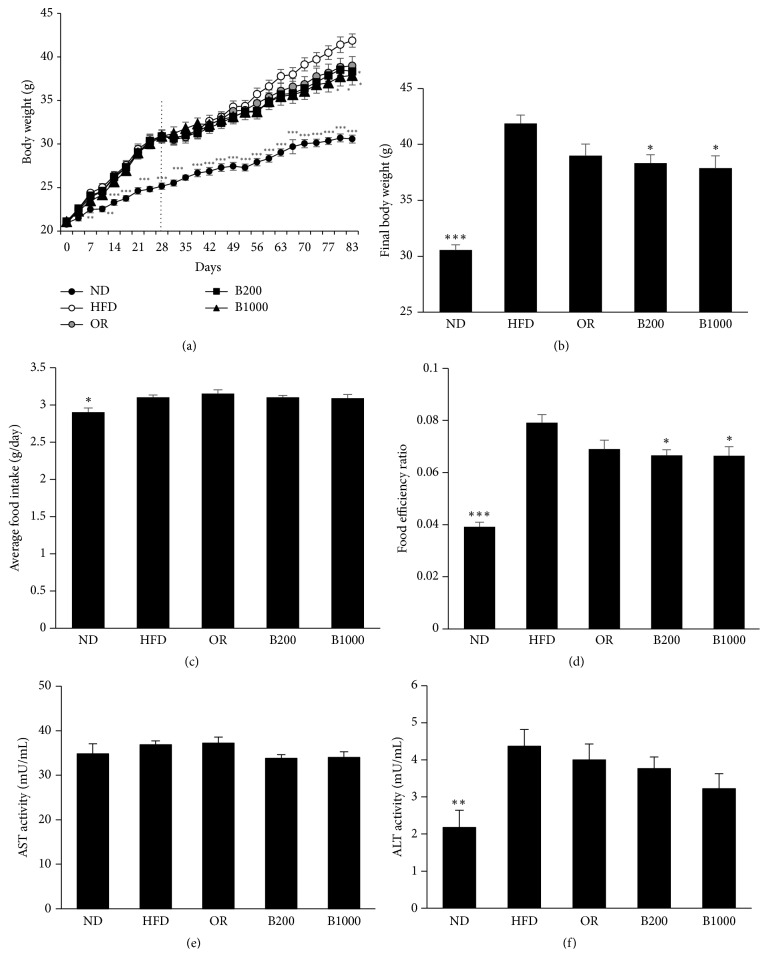
Effect of BHSST on (a) body weight change, (b) final body weight, (c) average food intake, (d) food efficiency ratio, (e) plasma AST activity, and (f) plasma ALT activity in diet-induced obese mice. Data are presented as the means ± SEM. Values with the different superscript letters indicate statistical significance (^*∗*^*p* < 0.05, ^*∗∗*^*p* < 0.01, and ^*∗∗∗*^*p* < 0.001) between groups. ND: normal diet-fed group; HFD: high-fat diet-fed group; OR: HFD with 50 mg/kg of Orlistat; B200: HFD-fed group with 200 mg/kg of BHSST; and B1000: HFD-fed group with 1000 mg/kg of BHSST.

**Figure 5:**
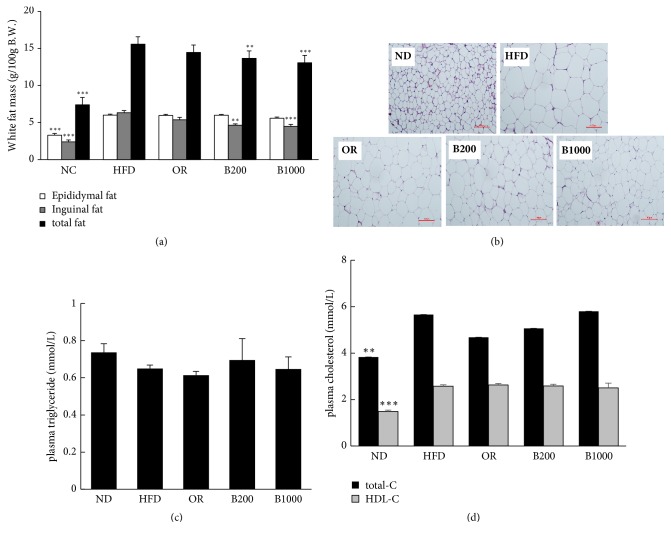
Effect of BHSST on (a) white fat mass, (b) histopathological analysis of inguinal fat (at 100 x of magnification), (c) plasma triglyceride level, and (d) plasma total cholesterol level in diet-induced obese mice. Data are presented as the means ± SEM. Values with the different superscript letters indicate statistical significance (^*∗∗*^*p* < 0.01 and ^*∗∗∗*^*p* < 0.001) between groups. ND: normal diet-fed group; HFD: high-fat diet-fed group; OR:, HFD with 50 mg/kg of Orlistat; B200: HFD-fed group with 200 mg/kg of BHSST; and B1000: HFD-fed group with 1000 mg/kg of BHSST.

After inducing obesity for 28 days, HFD significantly increased body weight from 30.57 to 41.87 g compared with ND (Figure 4(a)). However, BHSST, at both doses, prevented body weight gain. The body weight was markedly decreased in mice administered BHSST at 200 mg/kg after 77 days and at 1000 mg/kg after 83 days (Figures 4(a) and 4(b)). Consistently, HFD feeding significantly increased total white fat mass from 7.383 to 15.571 g/100 g body weight (Figure 5(a)). In contrast, BHSST administration decreased total white fat and inguinal fat mass compared with that in the HFD-fed group. Histological analysis of inguinal fat showed that the adipocytes size was markedly increased in the HFD-fed group versus that in the ND-fed group but reduced in the BHSST-treated groups (Figure 5(b)). Although BHSST did not affect food intake, it did decrease the food efficiency ratio (FER) (Figure 4(c)). Orlistat had no significant effect on the body weight, fat weight, food intake, and FER in the HFD-fed group (Figures 4 and 5). AST and ALT were unchanged by BHSST administration (Figures 4(e) and 4(f)).

The plasma cholesterol level was elevated in the HFD-fed groups compared with that in the ND-fed group, but the plasma TG level was not changed (Figures 5(c) and 5(d)). BHSST and Orlistat did not change the plasma levels of cholesterol and TG.

## 4. Discussion

Many studies have reported the various effects of medicinal herbs and their components. In the clinic, a mixture of several herbs, known as herbal formula, has primarily been used, rather than a single herb. 52 herbal formulas frequently used in traditional medicine were assessed the inhibitory effect of adipogenesis using 3T3-L1 adipocytes, BHSST was selected as one of the effective formulas to prevent triglyceride accumulation during adipogenesis. Previous study analyzed nine marker compounds—baicalin, wogonoside, baicalein, wogonin, glycyrrhizin, liquiritin, berberine, coptisine, and palmatine—in the BHSST extract using HPLC-PDA [9]. Baicalin [19], baicalein [20], glycyrrhizin [21], berberine [22, 23], coptisine [24], and palmatine [24] have inhibitory effect of obesity in* in vitro* or* in vivo* study. In particular, Baicalin, the most abundant component in BHSST, was well known for having antiobesity effects. Thus, we predicted that BHSST would exert antiobesity effect. In the present study, we investigated the inhibitory effect of BHSST on antiadipogenic effect using 3T3-L1 adipocytes and weight gain in obese mouse models. Furthermore, we evaluated the acute toxicity of BHSST in SD rats.

Adipose tissue is an important endocrine organ for lipid storage and energy homeostasis. The well-established preadipose cell line, 3T3-L1, responds in the same manner following treatment under experimental conditions [25]. Thus, we assessed whether BHSST affected adipocyte differentiation by using 3T3-L1 cells. TG accumulation was increased with the progression of 3T3-L1 cell differentiation. However, BHSST treatment reduced the intracellular TG content and GPDH activity, which is the first step in triglyceride synthesis [26]. Adipocyte differentiation is multistep process involving various transcription factors and extracellular proteins. The main transcription factors, PPAR-*γ* and C/EBP-*α*, act cooperatively to regulate the expression of target genes leading to adipocyte differentiation. Activated PPAR-*γ* triggers the downstream target genes involved in the differentiation program and induces positive feedback with C/EBP-*α* to sustain fat formation [27]. In the present study, BHSST treatment considerably downregulated the mRNA and protein levels of PPAR-*γ* and C/EBP-*α*. Consistently, BHSST downregulated mRNA and protein levels of FABP4 which is the major target gene of PPAR-*γ*.

The use of herbal medicine is gaining in popularity, but its safety is still controversial. Thus, it is necessary to evaluate the toxicity of herbal medicine to establish its safety and overcome its limitations. Before testing the antiobesity effect of BHSST, we evaluated the acute toxicity of BHSST through single oral administration. During the experimental period, there were no abnormal changes in the mortality, body weight, and gross findings after BHSST administration. Thus, the LD_50_ of BHSST was considered higher than 5000 mg/kg, regardless of sex.

Although natural products, including herbal formulas, are considered safe, there are a number of concerns regarding liver toxicity. Elevated levels of ALT and AST generally reflect hepatic damage [28]. During the administration of BHSST for 8 weeks in C57BL/6J mice, there was no significant change in ALT and AST levels. Orlistat, used as a positive control, is only one drug for long-term treatment of obesity. But the weight-loss effect of Orlistat is mild [29, 30]. In the present study, Orlistat did not affect the body weight and white adipose tissue weight in HFD-induced obese mice. Food intake is closely correlated with body weight gain based on a given diet. Thus, the FER was used as an index of the relative ability of a given food source to contribute to weight gain. Although food intake was similar in HFD-fed groups, final body weight and FER were significantly lower in BHSST-treated groups. Weight gain leads to an increase adipocyte size, and enlarged adipocytes have altered metabolic parameters [31]. The administration of BHSST with HFD for 8 weeks markedly reduced body weight as well as inguinal and adipocyte size.

## 5. Conclusions

In the present study, we used a traditional herbal formula, BHSST, which inhibited fat accumulation in adipose tissue with nontoxic effects. Thus, BHSST might be a helpful medication for weight management, but further studies are required to obtain information for safe usage. Further investigations, including a chronic toxicity study and clinical study, are needed to establish the safety and antiobesity drug development of BHSST.

## Figures and Tables

**Table 1:** Composition of Banhasasim-tang.

Latin name	Scientific name	Amount (g)	Origin
Pinelliae Tuber	*Pinellia ternata*	7.500	China
Scutellariae Radix	*Scutellaria baicalensis*	5.625	Korea
Ginseng Radix	*Panax ginseng*	5.625	Korea
Glycyrrhizae Radix et Rhizoma	*Glycyrrhiza uralensis*	5.625	China
Zingiberis Rhizoma	*Zingiber officinale*	3.750	Korea
Zingiberis Rhizoma Crudus	*Zingiber officinale*	3.750	Korea
Zizyphi Fructus	*Ziziphus jujuba*	3.750	Korea
Coptidis Rhizoma	*Coptis japonica*	1.875	China
Total		37.50	

**Table 2:** Mortality, gross findings, and clinical signs in rat single oral administrated with BHSST.

Group	Days on test^a^				Gross findings^b^	Loss of fur^c^
	1 day	≤3 Days	≤7 Days	≤15 Days		
Male						
0 mg/kg	0/5	0/5	0/5	0/5	0/5	0/5
5000 mg/kg	0/5	0/5	0/5	0/5	0/5	0/5
Female						
0 mg/kg	0/5	0/5	0/5	0/5	0/5	1/5
5000 mg/kg	0/5	0/5	0/5	0/5	0/5	0/5

^a^Number of animals with dead animals/total animal number.

^b,c^Number of animals with sign animals/total animal number.

## Data Availability

The data used to support the findings of this study are available from the corresponding author upon request.
